# Toxic Effects of Nickel Oxide Bulk and Nanoparticles on the Aquatic Plant *Lemna gibba* L.

**DOI:** 10.1155/2015/501326

**Published:** 2015-05-17

**Authors:** Abdallah Oukarroum, Lotfi Barhoumi, Mahshid Samadani, David Dewez

**Affiliations:** ^1^Département de Chimie, Université du Québec à Montréal, CP 8888, Succursale Centre-Ville, Montréal, QC, Canada H3C 3P8; ^2^Faculté des Sciences de Bizerte, Université de Carthage, Jarzouna, 7021 Bizerte, Tunisia

## Abstract

The aquatic plant *Lemna gibba* L. was used to investigate and compare the toxicity induced by 30 nm nickel oxide nanoparticles (NiO-NPs) and nickel(II) oxide as bulk (NiO-Bulk). Plants were exposed during 24 h to 0–1000 mg/L of NiO-NPs or NiO-Bulk. Analysis of physicochemical characteristics of nanoparticles in solution indicated agglomerations of NiO-NPs in culture medium and a wide size distribution was observed. Both NiO-NPs and NiO-Bulk caused a strong increase in reactive oxygen species (ROS) formation, especially at high concentration (1000 mg/L). These results showed a strong evidence of a cellular oxidative stress induction caused by the exposure to NiO. Under this condition, NiO-NPs and NiO-Bulk induced a strong inhibitory effect on the PSII quantum yield, indicating an alteration of the photosynthetic electron transport performance. Under the experimental conditions used, it is clear that the observed toxicity impact was mainly due to NiO particles effect. Therefore, results of this study permitted determining the use of ROS production as an early biomarker of NiO exposure on the aquatic plant model *L. gibba* used in toxicity testing.

## 1. Introduction

The specific properties of engineered metallic nanoparticles (NPs) are directly related to their nanosize, chemical composition, shape, and surface charge which permitted an extensive range of application in nanotechnology industries. However, these NPs received recently considerable attention in order to manage their safe use and several studies concerning the characterization of NPs demonstrated potential cytotoxicity for many aquatic organisms by causing a cellular oxidative stress effect [1–3]. Indeed, it was suggested for the toxicity mechanism and intensity of NPs suspension to be highly dependent on their physicochemical proprieties in aqueous solution. Several studies showed that the toxicity of NPs was directly related to their intrinsic properties of material itself, such as their relative hydrodynamic size, concentration, surface chemistry, shape, and the chemical characteristics of the exposure medium (e.g., pH and ionic strength) [4–7]. Several studies reported that the toxicity mechanism was related to the release of ions from nanoparticles and/or their direct interaction to membranes causing inhibitory effects on cellular functions [8–10]. However, others studies suggested that their toxic effects were directly related to NPs penetration and oxidation into the cellular system [11–13]. These NPs will inevitably be released into aquatic ecosystems through wastewater output, altering water quality and representing a risk of toxicity for freshwater organisms. Particularly, the effects of NPs on aquatic plants have been widely discussed in recent years since they are ecologically at the food base of trophic chains in the ecosystem [1, 14]. Therefore, the exposition of NPs in aquatic ecosystems represents a biological risk of toxicity which needs to be evaluated at cellular level for aquatic plants by considering the bioavailability and the uptake of NPs in aqueous solution.

Nickel oxide nanoparticles (NiO-NPs) possess unique properties compared to its bulk Nickel(II) oxide (NiO-Bulk), which can be used in industrial products promoting innovative applications [15]. Recently, it has been reportedthat NiO-NPs were able to be easily transported into biological systems inducing both cytotoxic and genotoxic effects [16]. However, the bioaccumulation effect of NiO-NPs on aquatic plant system has not been extensively examined, since only very few data are available concerning their toxicity.

In this toxicological study, we used the duckweed* Lemna gibba* L. as a model organism because of its rapid growth, small size, and floating leaves having a high capacity to uptake contaminants. Recently, duckweeds were widely used for the testing of substances based on growth inhibition [17], since they were known for a long time to be widespread aquatic macrophytes as a source of food for many aquatic organisms of higher trophic level [18]. Therefore, the aim of this work was to evaluate and compare the toxic effects of NiO-NPs and NiO-bulk by using* Lemna gibba* as an aquatic bioindicator. Particularly, we investigated the induction of ROS and the change of the photosynthetic activity when plants were exposed during 24 h to a range of concentrations between 0 and 1000 mg/L. Therefore, we were able to determine the use of the production of ROS as an early biomarker of NiO exposure before the deterioration of photosynthesis and plant growth. Finally, this study permitted defining the toxicity risk of NiO-NPs suspensions and NiO-bulk on the viability of duckweeds.

## 2. Material and Methods

### 2.1. Biological Material

The aquatic plant* Lemna gibba *L. was obtained from the Canadian Phycological Culture Centre (formerly UTCC #310). Plants were grown in an inorganic culture medium according to the Organisation for Economic Cooperation and Development (OECD) guidelines (AAP growth medium). This nutritive medium consisted of the following salts: NaNO_3_, 510 mg/L; MgCl_2_·6H_2_O, 240 mg/L; CaCl_2_·2H_2_O, 90 mg/L; MgSO_4_·7H_2_O, 290 mg/L; K_2_HPO_4_·3H_2_O, 30 mg/L; H_3_BO_3_, 3.7 mg/L; MnCl_2_·4H_2_O, 8.3 mg/L; FeCl_3_·6H_2_O, 3.2 mg/L; Na_2_EDTA·2H_2_O, 6.0 mg/L; ZnCl_2_, 66 *μ*g/L CoCl_2_·6H_2_O, 29 *μ*g/L; Na_2_MoO_4_·2H_2_O, 145 *μ*g/L; CuCl_2_·2H_2_O, 0.24 *μ*g/L; NaHCO_3_, 300 mg/L. Before the medium was autoclaved for sterilization, its pH was adjusted to 6.5 ± 0.1 using 0.1 M HCl. Ionic strength for* L. gibba* culture medium was 4.25 10^−3^ and was calculated with chemical equilibrium model software Visual MINTEQ 3.0.

Experiments were done in a growing chamber CONVIRON (Controlled Environments Limited, Winnipeg, MB, Canada) having a 16/8 h light/dark photoperiod. A light irradiance of 100 *μ*mol of photons m^−2^ s^−1^ was provided by cool white fluorescent lamps (Sylvania GRO-LUX F40/GS/WS).

### 2.2. Nickel Oxide Nanoparticles and Nickel(II) Oxide

Nickel(II) oxide was purchased from BDH Laboratory Supplies (UK) and it was used as NiO-Bulk. Nearly spherical nickel nanopowder (NiO-NPs) was purchased from MTI Corporation (Richmond, CA, USA). According to the manufacturer, the diameter of NiO-NPs was 30 nm, purity was 99.9%, and the specific surface area was 50–80 m^2^/g. In this study, NiO-NPs size distribution in* L. gibba* culture medium was determined by dynamic light scattering (DLS) with a ZetaPlus Particle Sizer (Brookhaven Instruments Corporation, USA) using 90Plus Particles Sizing Software Ver. 4.20. A stock suspension of 1000 mg/L was prepared and sonicated before use during 3 min with an ultrasonicator in order to homogenize the suspension of nanoparticles. Zeta potential in the culture media was determined by the electrophoretic mobility method with the ZetaPlus system. Measurements were done at 24 h of exposure in* L. gibba* medium under growth conditions. NiO-NPs were suspended in* L. gibba *culture medium to a concentration of 1 mg/mL, and absorbance spectra were measured by using a UV-VIS spectrophotometer (Lambda 40, Perkin Elmer). To determine the solubility of NiO-NPs, suspensions of 0–1000 mg/L were prepared and incubated for 24 h in the condition as described above for algal culture. NiO-NPs suspensions were then centrifuged at 12,000 g for 30 min. The supernatant was removed with care and filtered through cellulose filters (0.22 *μ*m) in order to remove before analysis any possible agglomerated nanoparticulate matter. The quantification of soluble Ni was done by atomic absorption spectrometry using a Varian SpectrAA 220 FS system.

### 2.3. Treatment Conditions

Triple-fronded* L. gibba *plants, in the exponential growth phase, were used for experiments. Five triple-fronded* L. gibba *plants in three replicates were exposed during 24 h in Petri dishes containing growth medium having initial NiO-NPs or NiO-Bulk concentrations of 0, 1, 10, 100, and 1000 mg/L under the same condition as described above.

### 2.4. Reactive Oxygen Species (ROS) Formation

The ROS formation was measured by using the cell permeable indicator 2′,7′dichlorodihydrofluorescein diacetate (H_2_DCFDA) [19]. Cellular esterases hydrolyze the probe to the nonfluorescent 2′,7′dichlorodihydrofluorescein (H_2_DCF), which is better retained in the cell. In the presence of ROS and cellular peroxidases, H_2_DCF is transformed to the highly fluorescent 2′,7′dichlorofluorescein (DCF). Plants were washed three times with half-strength Hutner's medium and treated with 5 *μ*M H_2_DCFDA for 30 min at 25°C [20]. Each treatment and control were treated with 5 *μ*M of H_2_DCFDA in 1 mL of solution. The DCF fluorescence signal was measured using an excitation wavelength at 485 nm and an emission wavelength at 530 nm. The DCF fluorescence was measured directly on intact plants and the fluorescence signal was normalized by fresh weight. All fluorescence data were collected using a fluorescence plate reader (SpectraMax M2e Multi-Mode Microplate Reader).

### 2.5. Chl *a* Fluorescence Transients

Chl *a* fluorescence measurements were conducted at room temperature with a portable fluorimeter “Plant Efficiency Analyzer” (Handy-PEA, Hansatech Instruments Ltd., King's Lynn Norfolk, UK). All* L. gibba* plants were dark adapted for at least 15 min before the measurements were started to allow all PSII reaction centers to be in open state (reoxidized) and the electron transport chain to be fully oxidized. The measurement consisted of a single strong 1 s light pulse of 3000 *μ*mol of photons m^−2^ s^−1^ and excitation intensity sufficient to ensure closure of all PSII reaction centers, which was provided by an array of three ultrabright red light-emitting diodes (peak at 650 nm) in the instrument.

### 2.6. Fluorescence Parameters

Based on the fluorescence transients measured during the first second of illumination, several biophysical expressions leading to a description of a photosynthetic sample in a given physiological state were evaluated. One of the parameters calculated is the maximum yield of primary photochemistry of PSII (*F*_*V*_/*F*_*M*_). It corresponds to the efficiency by which an absorbed photon will be trapped by PSII reaction centers [21]. The absorption of photons (ABS) per active reaction center (RC) showing the antenna size was estimated by the ratio ABS/RC. The performance index of PSII activity (PI), which consider all PSII photochemical reactions, was one of the Chl *a* fluorescence parameters providing useful and quantitative information about the complete state of photosynthesis and the organism vitality. The formula expression of the performance index of PSII activity was well explained in reference [22].

### 2.7. Data Analysis and Statistics

For all treatments, means were determined for each treatment condition. Significant differences between control and treated samples were determined by using one-way analysis of variance (ANOVA) and multiple comparison of Tukey's test, where *P* values less than 0.05 (*P* < 0.05) were considered to be significantly different.

## 3. Results and Discussion

**Figure 1:**
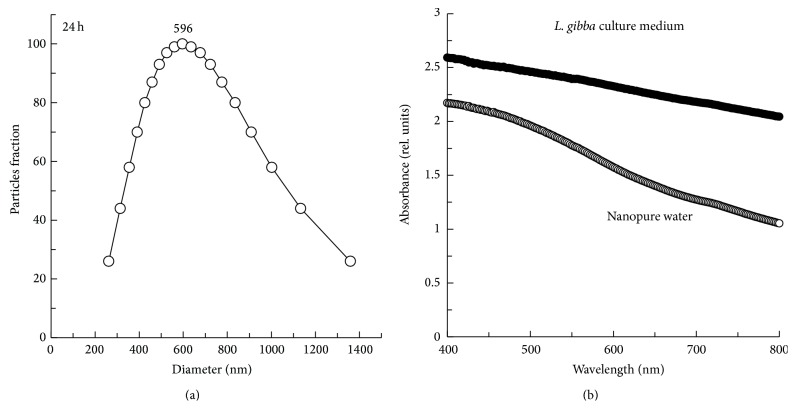
(a) Size distribution of NiO-NPs suspensions prepared in culture medium of* L. gibba* and measured at 24 h of incubation. (b) Absorbance spectra of NiO-NPs suspensions in* L. gibba* culture medium (1 mg/mL).

Since metallic nanoparticles represent hazardous aquatic contaminants, the toxic potential of NiO-NPs needs to be determined for the development of bioassays used in toxicity risk assessment. In this regard, the aquatic plant* L. gibba* represents a sensitive organism in toxicity testing. In this toxicological investigation, we clearly showed the potential source of toxicity of NiO-NPs suspensions in comparison to NiO-Bulk. Indeed, nickel(II) oxide is known to be practically insoluble in water limiting considerably the release of free ionic Ni and its bioavailability to aquatic organisms. On the other hand, the physicochemical conditions of the aqueous solution may alter the properties of NiO-NPs, modifying their bioavailability by changing their surface of contact, charge, and solubility. Particle size measurements showed that NiO-NPs formed rapidly a wide size distribution of agglomerations when suspended in* L. gibba* culture medium, which was found to be stable in the culture medium during the entire experimental exposure (Figure 1). The median diameter of particle size distribution for nanoparticles suspension in culture medium was 596 nm. Such agglomerations were formed due to physicochemical properties of the* L. gibba* culture medium, such as the pH of 6.5 and the composition of nutrients causing an ionic strength of 4.25 × 10^−3^. Such conditions were affecting the interaction of NiO-NPs with inorganic ions by changing the surface charge of nanoparticles.Here, we noted that the charge at NiO-NPs surface was negative in* L. gibba* culture medium, equalling −26.81 (±0.5). Indeed, it has been previously reported that nanoparticles were forming agglomerates in testing media by changing the surface charge of nanoparticles due to ionic strength [7, 14, 23, 24]. Moreover, the formation of agglomerates was affecting the solubility of NiO-NPs in* L. gibba* culture medium since our results indicated very low concentrations of ionic form of nickel in the soluble fraction in comparison to total mass of nanoparticles (Table 1). Such low solubility of NiO-NPs was also observed when they were suspended in other culture media [25, 26]. Indeed, the formation of agglomerates caused their sedimentation at the bottom of the experimental flask, decreasing their surface contact with the medium and their solubilization. Therefore, toxic effects of NiO-NPs need to be directly related to their physicochemical characteristics such as their solubility in the* L. gibba* culture medium.

**Figure 2:**
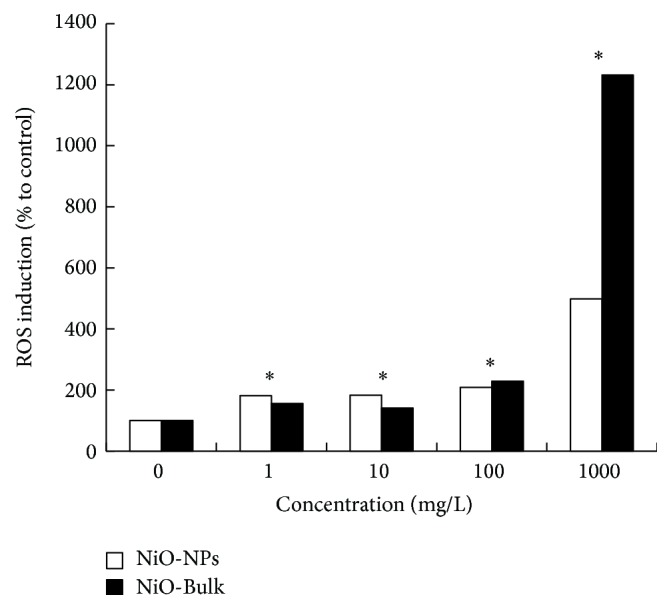
Change in the production of reactive oxygen species (ROS) for* L. gibba* plants exposed during 24 h to different concentrations of NiO-Bulk or NiO-NPs suspensions. Asterisks indicate statistical significance between control and treatment (*P* < 0.05).

For environmental protection agencies, the increase release of nanoparticles into the aquatic environment represents a risk for the integrity of the food web through their interactions with various microorganisms, invertebrates, and fish. With such concern, many studies showed the evidence of the bioaccumulation effects of metallic nanoparticles in aquatic organisms, in which the cytotoxicity impact was especially indicated by the induction of oxidative stress and the inhibition of growth [27]. In the present study, our bioassay of toxicity focused on the production of intracellular ROS using the plant model* L. gibba*. Indeed, the production of ROS was used as an early and rapid biomarker of the presence of NiO-NPs cytotoxicity already at 24 h of treatment. It is important to notice that the visible spectrum of NiO-NPs (in Figure 1) showed no specific band at 530 nm, suggesting that the ROS formation was mainly due to the highly fluorescent compound 2′,7′-dichlorofluorescein (DCF). Our results indicated a significant increase in intracellular ROS concentrations when* L. gibba* plants were exposed to NiO-NPs suspensions (Figure 2). Particularly, the ROS formation increased by 90% compared to control (*P* < 0.05) at the lowest concentration of NiO-NPs. Moreover, the strongest fluorescence signal of the ROS sensor (H_2_DCFDA) was found for* L. gibba* plant exposed to 1000 mg/L, indicating an increased production of ROS by 5 times compared to control (*P* < 0.05). Concerning the effect of NiO-Bulk, the production of ROS was similar to NiO-NPs under the testing exposure concentration range of 1–100 mg/L. However, when* L. gibba* plant was exposed to 1000 mg/L of NiO-Bulk or NiO-NPs, the production of ROS was 2 times higher for NiO-Bulk in comparison to NiO-NPs. These results clearly indicated that the induction of oxidative stress toxicity was not directly related to NiO-NPs solubility. Therefore, NiO-NPs were uptaken into* L. gibba* plant system causing the production of intracellular ROS, directly through photocatalysis or indirectly through molecular cell damages. In an earlier study, researchers explored the bioaccumulation effects of NiO-NPs on algal cells of* Chlorella vulgaris* [26]. They observed that NiO-NPs formed deposits in the cytoplasm which provoked growth inhibition and cellular ultrastructure alterations after 72 h of exposure to 10–50 mg/L of NiO-NPs. However, our results indicated that NiO-NPs possess a different toxicity mechanism in comparison to other metallic nanoparticles, for which it was suggested that their solubilization into free metal ions was the main mechanism of toxicity [10, 28].

**Figure 3:**
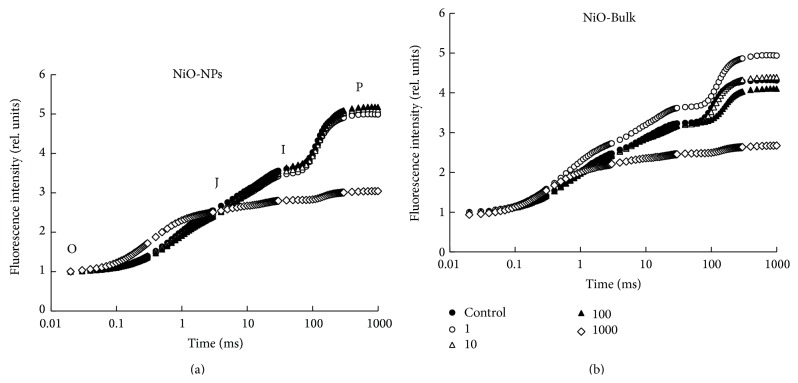
Fast Chl *a* fluorescence OJIP transients exhibited when* L. gibba* plants were exposed to 0, 1, 10, 100, and 1000 mg/L of NiO-Bulk or NiO-NPs suspensions (*n* = 5). The symbols used are O, J, I, and P representing, respectively, the fluorescence intensities at 50 *μ*s, 2 ms, 30 ms, and 200 ms.

**Figure 4:**
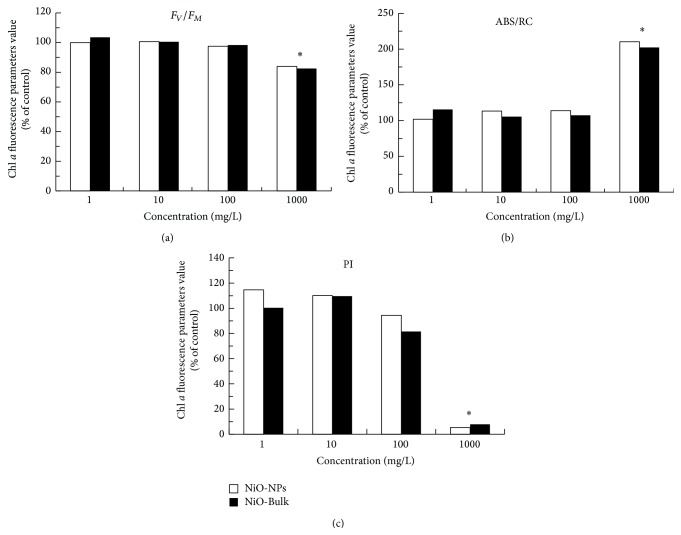
Change in the Chl *a* fluorescence parameters such as the maximum quantum yield of primary photochemistry (*F*_*V*_/*F*_*M*_) and the ratio between light-harvesting chlorophyll antenna size and the number of active PSII reaction centers (ABS/RC) and the performance index of PSII activity (PI) for* L. gibba* plants exposed during 24 h to different concentrations of NiO-Bulk or NiO-NPs suspensions (*n* = 5).

Moreover, the toxic effect of NiO-NPs was investigated on the photosynthetic functions and compared to NiO-Bulk effects. The chlorophyll fluorescence rise measured from all leaves displayed the typical OJIP transients when plotted on a logarithmic time scale (Figure 3). The fluorescence transients showed two steps between O and P, the J transient at about 2 ms and the I transient at 30 ms. It was previously demonstrated that the O-J phase was strongly light dependent on the reduction of quinone Q_A_ [22] and the J-P rise reflected the reduction of the rest of the electron transport chain after Q_A_ [29]. When* L. gibba* plants were exposed to 1000 mg/L of NiO-NPs or NiO-Bulk effects, it was noticed that the induction of the OJIP fluorescence transients differed significantly in comparison to control plants (Figure 3). Under this experimental condition, results demonstrated an inhibitory effect of NiO-NPs and NiO-Bulk on the photochemical activity of the photosystem II (PSII) which was indicated by the reduction in the quantum yield of PSII electron transport (Figure 4). This alteration suggested damages to the structural and functional properties of PSII. Furthermore, the increase of ABS/RC ratio indicated an increase in the inactivation of PSII reaction centers which was related to a downregulation mechanism to dissipate the excess of excitation energy from chlorophyll molecules (Figure 4). Similarly, the reduction of the overall process of PSII photochemistry and electron transport activity, shown by the decrease in PI parameter value, indicated that both NiO-NPs and NiO-Bulk were able to alter directly the photosynthetic performance of* L. gibba* plant.

Previously, an* in vitro* study on PSII submembrane fractions isolated from spinach showed that NiCl_2_ at millimolar range of concentration had a strong inhibitory effect on PSII reaction center functions and oxygen evolution activity [30]. In another study, using two duckweed species,* Spirodela polyrhiza* clone SJ and* Lemna minor* clone St, it was shown that NiCl_2_ (the most soluble form of Ni) induced a strong decrease in the contents of both chlorophylls *a* and *b* and also an inhibition of fresh weight over a 7-day test period [31]. However, our toxicological investigation indicated that over a 24 h toxicity testing period, the toxicity impact of NiO was not induced by the free ionic form of Ni. Nevertheless, under a longer time of treatment such as 7 days, it is possible that the soluble fraction containing free ionic Ni may also contribute to the cellular toxic effects.

In this study, we clearly showed the potential source of toxicity of NiO-NPs suspensions in comparison to NiO-Bulk. The aquatic plant* Lemna gibba* demonstrated to be a valuable bioindicator of NiO cellular toxicity, which was indicated by the deterioration of photochemical activities of photosynthesis and the induction of oxidative stress. Under the experimental conditions used, it is clear that the observed toxicity impact was mainly due to NiO particles effect. Therefore, results of this study permitted determining the use of ROS production as an early toxicity biomarker of NiO exposure to* L. gibba* plant.

## Figures and Tables

**Table 1:** Change in dissolved free ionic nickel (in mg/L) released from different concentrations of NiO-NPs suspensions in *L. gibba* culture medium at 24 h of treatment.

[NiO-NPs] mg/L	[Free ionic Ni] mg/L
1	n/a
10	0.0510 ± 0.05
100	1.10 ± 0.6
1000	5.93 ± 0.2
